# PDE4 Inhibitors and their Potential Combinations for the Treatment of Chronic Obstructive Pulmonary Disease: A Narrative Review

**DOI:** 10.2174/0118743064340418241021095046

**Published:** 2024-11-13

**Authors:** Rakesh Kumar, Mohd Imran Khan, Amit Panwar, Bhavishya Vashist, Santosh Kumar Rai, Anil Kumar

**Affiliations:** 1 New Drug Discovery Research, Mankind Research Centre, Mankind Pharma Limited, Plot No 191-E, Sector 4-II, IMT Manesar, Gurugram, India-122051

**Keywords:** COPD, Bronchodilators, ICS, PDE4 inhibitors, Dual PDE4/PDE3 inhibitors, Anti-inflammatory

## Abstract

Chronic Obstructive Pulmonary Disease (COPD) is associated with cough, sputum production, and a reduction in lung function, quality of life, and life expectancy. Currently, bronchodilator combinations (β2-agonists and muscarinic receptor antagonists, dual therapy) and bronchodilators combined with inhaled corticosteroids (ICS), triple therapy, are the mainstays for the management of COPD. However, the use of ICS in triple therapy has been shown to increase the risk of pneumonia in some patients. These findings have laid the foundation for developing new therapies that possess both anti-inflammatory and/or bronchodilation properties. Phosphodiesterase-4 (PDE4) inhibitors have been reported as an effective therapeutic strategy for inflammatory conditions, such as asthma and COPD, but their use is limited because of class-related side effects. Efforts have been made to mitigate these side effects by targeting the PDE4B subtype of PDE4, which plays a pivotal role in the anti-inflammatory effects. Unfortunately, no selective oral PDE4B inhibitors have progressed to clinical trials. This has led to the development of inhaled PDE4 inhibitors to minimize systemic exposure and maximize the therapeutic effect. Another approach, the bronchodilation property of PDE3 inhibitors, is combined with anti-inflammatory PDE4 inhibitors to develop dual inhaled PDE4/PDE3 inhibitors. A few of these dual inhibitors have shown positive effects and are in phase 3 studies. The current review provides an overview of various PDE4 inhibitors in the treatment of COPD. The possibility of studying different selective PDE4 inhibitors and dual PDE3/4 inhibitors in combination with currently available treatments as a way forward to increase their therapeutic effectiveness is also emphasized.

## 1 INTRODUCTION

Chronic Obstructive Pulmonary Disease (COPD) is a common and treatable disease that is characterized by an abnormal inflammatory response in the lungs [1]. Around 11.7% of the global population is estimated to be affected by COPD, although these figures could be underestimated due to the rapid increase in COPD prevalence [2-4]. According to the Fixed Ratio (FR) criteria, the highest COPD prevalence was found in the American region (22.93%), followed by the Southeast Asian region (19.48%), which was followed by Europe (13.09%), Western Pacific (11.17%), and Eastern Mediterranean regions (7.95%) [5]. The prevalence of COPD was 10.43% in the 2016-2019 period and increased to 15.17% in the 2020-2022 period [5]. Although COPD can be prevented and treated, it is still among the top 10 causes of mortality worldwide [6], the sixth leading cause of death in the United States [7], and is a major reason for morbidity and healthcare expenditures. COPD is predicted to be the leading cause of death worldwide in future years [8].

COPD is considered a systemic disease, which is more common in individuals with a history of tobacco smoking [5, 8]. Smoking tobacco is the most common contributing factor in developed and developing countries [5, 9, 10]. Besides smoking, various comorbidities and risk factors are reported to be associated with this disease, including genetics, infections, malnutrition, aging, occupational exposures, indoor and outdoor air pollutants, asthma, and low socioeconomic status [8, 10]. In COPD, a large population of patients suffers from exacerbations, which are described as an acute worsening of respiratory signs. Severe exacerbations are always related to a significantly worse survival outcome [11].

It is estimated that COPD contributes approximately $40 billion annually in US healthcare expenditures [12]. Most of the contributing factors to the economic burden of COPD are frequent or severe exacerbations, which require frequent visits to the emergency department or may require hospitalization. Hence, current objectives to treat COPD patients should be changed towards symptom reduction, exacerbation prevention, and reducing the risk of premature death.

The Global Initiative for Chronic Obstructive Lung Disease (GOLD, 2023) has recommended maintenance therapy based on COPD severity, symptoms assessment, and exacerbation history [13]. Long-acting bron- chodilators, such as Long-Acting Muscarinic Antagonists (LAMAs) and long-acting β2-agonists (LABAs), are the basis of maintenance therapy for COPD patients (Table **1**) [13, 14].

LAMA or LABA monotherapy has not always been found satisfactory, while combination therapy has been reported to have superior effects (Table **1**) [15]. The combination of LABAs and LAMAs maximized bronchodilation with an insignificant increase in the side effects compared to increasing the dose of a single bronchodilator [13]. Since the COPD mechanism is related to inflammation, treatments like Inhaled Corticosteroids (ICSs) have been evaluated in combination with existing therapies. The addition of an Inhaled Corticosteroid (ICS) to LABAs and/or LAMAs is recommended for patients with frequent exacerbations and high blood eosinophil levels [13, 16]. The recommended triple combination therapies for the treatment of COPD are mentioned in (Table **2**).

Inflammatory diseases like asthma, COPD, rheumatoid arthritis (RA), or psoriasis can be treated by inhibiting phosphodiesterase-4 (PDE4) [17]. The PDE4 inhibition suppresses airway inflammation and relaxes smooth muscle *via* an elevation of cAMP (cyclic adenosine monophosphate) levels [17]. Roflumilast is the only PDE4 inhibitor approved for the treatment of patients with severe COPD. A major problem with oral PDE4 inhibitors is the mechanism-based side effects, *i.e*., nausea, emesis, diarrhea, and headache, which have restricted their use to lower doses only [18]. Thus, there is still considerable room for the development of more efficacious and safe PDE inhibitors for the treatment of asthma and COPD. To date, different strategies have been adopted to enhance the efficacy and safety of PDE4 inhibitors, as mentioned below [18]:

(1) One of the approaches to increase the efficacy of oral PDE4 inhibitors is to selectively target one of the PDE4 subtypes, PDE4B, which is responsible for most of the anti-inflammatory effects [19]. A PDE4D selective inhibitor, like cilomilast, caused emesis and demonstrated a poor therapeutic index compared to the non-selective roflumilast and cilomilast [20-22].

(2) Another approach could be the development of inhaled PDE4 inhibitor formulations to deliver them directly to the lungs in order to avoid systemic exposure. Among several inhaled PDE4 inhibitors, a few have progressed to clinical trials for the treatment of asthma and COPD [18, 23-27].

(3) PDE3 inhibition causes bronchodilation, and selective PDE4 inhibitors are anti-inflammatory but lack acute bronchodilator activity [28, 29]. Therefore, several researchers have tried to develop dual inhibitors of PDE3 and PDE4 to achieve cumulative effects. It has also been proposed that dual PDE3/PDE4 inhibitors may enhance the effects of LABA/ICS combination therapies in asthma or COPD patients [30, 31].

(4) Another approach is to study PDE4 inhibitors in combination with available bronchodilators (LABA/LAMA) or triple therapy for COPD. Roflumilast has demonstrated improvement in lung function in patients with asthma or COPD when combined with ICS or LABA/LAMA [30, 31].

(5) Based on the above-mentioned different app- roaches to improve the therapeutic index, this review lays out future goals for research focused on the basis of efficacy and safety of PDE4 inhibitor combinations/FDC (Fixed-Dose Combination) and dual inhibitors of PDE3/4 as drugs for the treatment of patients with asthma or COPD.

### 1.1 Chronic Inflammation in COPD and the Role of PDEs

Inflammation and structural modifications in the lungs are major causes of airflow limitation and the decline in forced expiratory volume in 1 second (FEV1) in COPD patients. Inflammatory cells like neutrophils, macro- phages, and T cells are mainly involved in COPD pathophysiology. Structural cells (*e.g*., epithelial cells, fibroblasts, and smooth muscle cells) also contribute by releasing cytokines, chemokines, and growth factors (*e.g*., IL-8 and TNF-α) [32, 33]. PDEs are divided into 11 families (PDE1 to PDE11) and are expressed in different cell types of the lungs [34]. PDEs hydrolyze cAMP and cGMP, and these second messengers play important roles in asthma or COPD by regulating the Airway Smooth Muscle (ASM) tone *via* the β2-adrenergic (β2-AR)-soluble adenylyl cyclase (sAC)-cAMP signaling pathway. A higher level of cAMP in ASM is responsible for its relaxation and prevents various inflammatory responses, which play a fundamental role in the pathophysiology of COPD [35]. cAMP-specific PDEs are PDE4, PDE7, and PDE8, while PDE5, PDE6, and PDE9 are cGMP-specific PDEs. PDE1, PDE2, PDE3, PDE10, and PDE11 can hydrolyze both cAMP and cGMP [31].

**Fig. (1):**
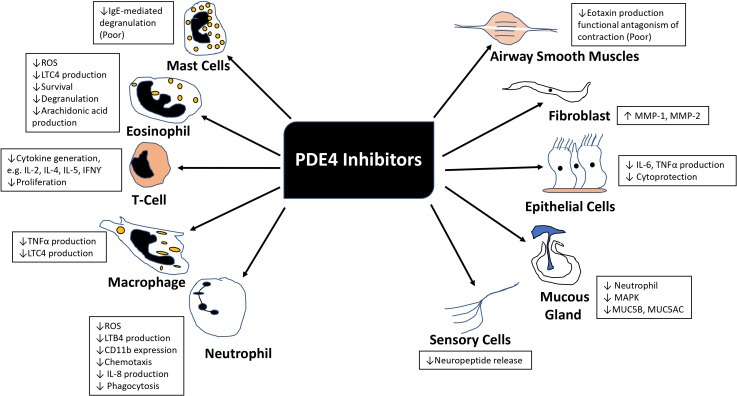
Anti-inflammatory effects of PDE4 inhibitors in COPD patients. (ROS, Reactive Oxygen Species; LTC4, Leukotriene C4; IL2, Interleukin-2; IL4, Interleukin-4; IL5, Interleukin-5; IL8, Interleukin-8; IFNγ, Interferon‐gamma; TNFα, Tumor necrosis factor alpha; LTB4, Leukotriene B4; MMP-1, Matrix metalloproteinase-1; MMP-2, Matrix metalloproteinase-2; MAPK, Mitogen-activated protein kinase; MUC5B, Mucin 5B; MUC5AC, Mucin 5AC).

All PDE4 isoenzymes are mainly expressed in T-cells, B-cells, eosinophils, neutrophils, airway epithelial cells, and endothelial cells [36]. PDE4 is also expressed in ASM cells, but its inhibition has not exhibited bronchodilator effects in humans [31]. Predominant PDE4 activity is also found in the ciliary epithelia [37]. The PDE4 family of enzymes has been gaining attention in asthma and COPD because PDE4 inhibitors have shown anti-inflammatory effects (Fig. **1**).

## 2 PDE4 INHIBITORS: MONOTHERAPY

### 2.1 Oral PDE4 Inhibitors

**Fig. (2):**
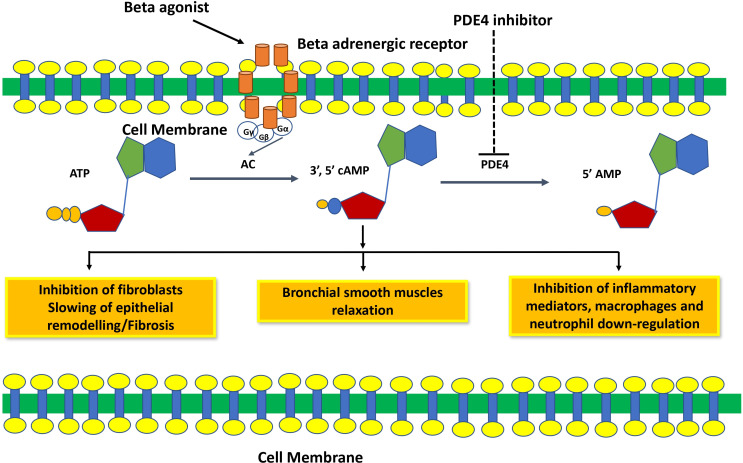
Mechanism of action of phosphodiesterase 4 inhibitors. PDE4 inhibitors inhibit the degradation of cAMP and increase cAMP levels, which relaxes smooth muscle and inhibits inflammation and fibrosis.

Theophylline or 1,3-dimethylxanthine inhibits PDE and is used to treat COPD or asthma. Being a PDE inhibitor, theophylline exerts anti-inflammatory and immuno- modulatory effects through inhibition of cAMP degra- dation [38-41], although the mechanism of action of theophylline is not fully understood [42]. Theophylline is not a selective PDE inhibitor and inhibits all PDEs in different tissues and organs, including the cardiovascular, gastrointestinal, and central nervous systems (Fig. **2**) [41, 42].

Roflumilast was approved for COPD patients suffering from severe airflow obstruction and a history of previous exacerbations, although it was associated with side effects [43]. Oral roflumilast was found to improve lung function and reduce the frequency of exacerbations in COPD patients in two large Phase 3 trials [44]. In a double-blind, placebo-controlled, multicentre, phase 4 trial (NCT013- 29029), roflumilast was tested to reduce exacerbations and hospitalizations in patients with severe chronic obstructive pulmonary disease and chronic bronchitis. It was reported that roflumilast decreased exacerbations by 13.2% lower than the placebo group; however, adverse events were observed in 67% of patients in the roflumilast group and 59% in the placebo group. Patient withdrawals related to adverse events were higher in the roflumilast group (11%) than in the placebo group (5%). The most commonly reported serious adverse events were exacerbations of chronic obstructive pulmonary disease and pneumonia, with 17 (1.8%) deaths in the roflumilast group *versus* 18 (1.9%) in the placebo group. Weight loss was higher in the roflumilast group (9%) compared to the placebo group (3%) [45].

Another orally available second-generation PDE4 inhibitor, Cilomilast, significantly improved lung function and quality of life in Phase I and Phase II clinical trials [46]. However, dose-limiting adverse effects were a major concern in the Phase III clinical study and attributed to interactions with PDE4 expressed in “non-target” tissues [46]. Higher side effects were produced by cilomilast in comparison to other systemically delivered PDE4 inhibitors due to selective inhibition of the PDE4D subtype [21]. Hence, a better therapeutic index of roflumilast was speculated because of its pan inhibition of all PDE4 subtypes by roflumilast [18].

### 2.2 PDE4 Subtypes (PDE4A-D) Selective Inhibitors

Serious side effects (nausea and headache) associated with most of the oral PDE4 inhibitors led to their elimination from clinical trials because of higher systemic exposure. The development of PDE4B subtype-specific inhibitors was one of the strategies adopted to avoid such side effects since these side effects are thought to be associated with the inhibition of the PDE4D isoenzyme [20].

Four isotypes of PDE4 are expressed by four genes (PDE4A-D), and each gene contains various transcripts to produce long, short, and super-short protein isoforms. Long forms of PDE4 contain two upstream regions, UCR1 and UCR2. Phosphorylation of UCR1 by Protein Kinase A (PKA) is known to form a negative regulatory module. Negative regulation of UCR2 results in closing the active site, hence preventing access to cAMP [47]. All PDE4 isoforms also contain a C-terminal Control Region (CR3) shown to close over the active site by weakly engaging inhibitors. PDE4B accounts for most of the anti-inflammatory effects [19], and PDE4D is related to emesis [20]; therefore, developing PDE4B-specific inhibitors could be a useful strategy to treat COPD. PDE4D and PDE4B are required for neutrophil recruitment to the lung after exposure to endotoxin [19]. A single amino acid polymorphism in CR3 is responsible for PDE4B selectivity, *i.e*., a leucine in place of glutamine in PDE4B-CR3 causes a 70-80-fold shift in selectivity for any inhibitor. A series of arylpyrimidine-based PDE4 inhibitors have been reported by Naganuma *et al*., which showed >100-fold selectivity for PDE4B over PDE4D [48]. Highly selective triazine-based PDE4B inhibitors have been developed and reported by Hagen *et al*. [49]. High similarity in catalytic domains of PDE4B and PDE4D made it difficult to design PDE4 subfamily selective inhibitors. Unfortunately, no oral PDE4B-selective inhibitor has progressed to clinical trials [31].

### 2.3 Inhaled PDE4 Inhibitors

**Fig. (3):**
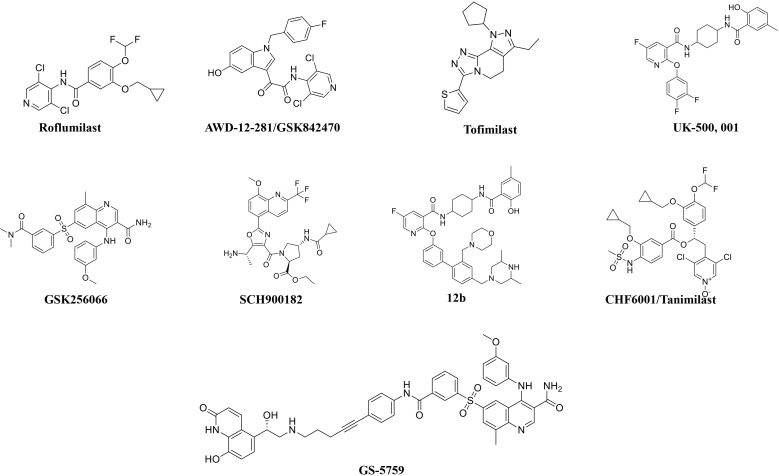
The chemical structures of roflumilast and inhaled PDE4 inhibitors.

Roflumilast use has been reduced because of adverse effects, such as nausea, diarrhea, weight loss, and abdominal pain, which led to significant treatment discontinuation and withdrawal from clinical trials. Higher systemic exposure to roflumilast caused these adverse effects and led to the development of inhaled PDE4 inhibitors to get maximum exposure in the lungs only [24-27, 50]. Several PDE4 inhibitors have been synthesized for inhaled administration, and very few of them have progressed to clinical trials for the treatment of asthma and COPD [18]. Several companies have disclosed data on inhaled PDE4 inhibitors (Fig. **3**). AWD-12-281 was a potent inhaled PDE4 inhibitor (IC_50_ = 9.7 nM) [51, 52] and showed preclinical efficacy in different species [53]. Poor efficacy led to the discontinuation of AWD-12-281. Tofimilast is a less potent (IC_50_ = 140 nM) inhaled PDE4 inhibitor [54] and has been found inefficacious in various clinical trials, *i.e*., in mild asthma [55], persistent asthma [56], GOLD stage II and III COPD patients, and LPS-challenged healthy subjects [57]. Therefore, the development of this compound was discontinued.

UK-500,001, an inhaled isotype-nonspecific but selective PDE-4 inhibitor (IC_50_ = 1 nM), was tested at three different doses in COPD patients. However, it did not demonstrate efficacy at any dose and was discontinued [18, 58]. GSK256066 (IC_50_ = 3 pM) is a PDE4B selective inhibitor and is claimed to be the most potent of all known PDE4 inhibitors [59, 60]. GSK256066 exhibited anti-inflammatory effects in LPS-stimulated human peripheral blood monocytes and whole blood, as well as in preclinical rat models of pulmonary inflammation [59, 60]. GSK256066 demonstrated a protective effect in mild asthma patients challenged with an inhaled allergen [61]. GSK256066 did not cause substantial changes in inflammatory markers, although it was well-tolerated in patients with moderate COPD [62].

Another effective PDE4 inhibitor is SCH900182 (IC_50_ = 0.07 nM), but this compound is in preclinical development and has not progressed to clinical studies [63, 64]. AstraZeneca has developed another inhaled PDE4 inhibitor, 12b (IC_50_ = 0.02 nM), with high potency [65]. 12b exhibited anti-inflammatory activity in the lungs of LPS-induced rats, and a very low predicted effective dose (1 μg/kg) may not cause emesis in humans [65]. Gilead synthesized a bifunctional compound, GS-5759, by linking a PDE4 inhibitor to a b2-agonist. This molecule moderately inhibited PDE4 (IC_50_ = 5 nM) [66, 67] and demonstrated *in-vivo* preclinical efficacy [68]. No clinical development has been reported for GS-5759. Two potent chemical series, the naphthyridinones (IC_50_ = 0.17 nM) [69] and pyridazinones (IC_50_ = 0.05 nM) [70], have been published, but compounds have not advanced to clinical development.

CHF 6001/Tanimilast is a highly potent but non-isotype selective PDE4 inhibitor (IC_50_ = 0.026 nM) and inhibited all isoforms (PDE4A-D) with equal potency [71]. CHF 6001 exhibited preclinical efficacy in various species and was also well tolerated in humans [71-73]. In a phase 2 clinical study, patients with COPD or chronic bronchitis (receiving inhaled triple therapy for ≥2 months) were treated with CHF6001 (800 or 1600 μg) twice daily *via* multi-dose dry powder inhaler for 32 days. This study confirmed that inhaled CHF6001 is capable of showing anti-inflammatory effects in the lungs of COPD patients already treated with triple inhaled therapy [74]. In another randomized, double-blind, placebo-controlled study, CHF6001 effects for moderate-to-severe exacerbations were non-significant in patients with a chronic bronchitis phenotype. These effects were further increased in patients with chronic bronchitis and eosinophil count ≥150 cells/μL [75, 76], indicating that a specific patient population (eosinophil count ≥150 cells/μL) may be more beneficial from CHF6001 treatment. Overall, both doses of CHF6001 were well-tolerated with no gastrointestinal adverse event (due to minimal systemic exposure) compared to placebo [74, 75]. These findings provided beneficial information for patients with chronic bronchitis, indicating that tanimilast could have additional beneficial effects in patients who are still symptomatic even after the regular use of ICS, LABA, and LAMA [57]. The benefits of tanimilast in this particular patient population are still under investigation in two large phase-III trials [PILASTER (NCT04636801); PILLAR (NCT04636814)] (Table **3**).

As discussed above, a significant effect of tanimilast was found in chronic bronchitis patients with higher eosinophil counts, where tanimilast reduced eosinophils and other key type-2 mediators in sputum [76, 77]. Also, a reduction in the exacerbation rate was significant in patients with chronic bronchitis and blood eosinophils ≥150 cells/μL [75]. This clinical data showed that the effect of tanimilast on type-2 inflammation is responsible for its clinical outcomes; however, further confirmation is needed [57].

### 2.4 Dual PDE4 and PDE3 Inhibitors

It has been well understood that a single molecule having bronchodilation and anti-inflammatory effects or a combination of a molecule having bronchodilation effects with an anti-inflammatory molecule could be a potential approach to dealing with COPD. However, selective PDE4 inhibitors (*e.g*., roflumilast) are anti-inflammatory but lack acute bronchodilation activity. Therefore, airway inflammation may not be resolved by targeting PDE4 alone. As already mentioned, different PDE isozymes selectively regulate cAMP or cGMP signaling, and PDEs are involved in specific locations at certain time points based on different stimulations/activations [34, 78, 79]. Hence, simultaneous targeting of different PDE enzymes with bifunctional drugs may be essential to have optimal anti-inflammatory and/or bronchodilation effects in asthma and COPD patients [80, 81].

Currently, particular emphasis has been given to the PDE3 since this isozyme can hydrolyze both cAMP and cGMP, with 5-10-fold higher catalytic rates for cAMP [82]. PDE3 is mainly expressed in airway smooth muscle, along with PDE4B and PDE4D [83]. PDE3 inhibition is responsible for bronchodilation through airway smooth muscle relaxation, whereas PDE4 inhibition is associated with an anti-inflammatory effect [27, 82]. Therefore, the development of dual PDE3/4 inhibitors could have better therapeutic effects in patients with COPD and asthma [80, 81, 84]. Very few dual PDE3/4 inhibitors have been published as well as reached in clinical trials to date (Table **4**).

Zardaverine is a 6-phenyl-2H-pyridazin-3-one, having IC_50_ values of 110 nM and 210 nM, respectively, for human PDE3 and PDE4 [85] and has shown bronchodilation [86, 87] and anti-inflammatory [88] activity in animal models. Clinical trials using oral and intravenous delivery demonstrated that zardaverine caused bronchodilation but also showed dose-limiting adverse effects, including emesis. Although the compound was better tolerated and retained bronchodilator efficacy by inhalation in asthmatics [89], inhaled zardaverine still did not cause lung function improvements in a trial in COPD patients [90]. Zardaverine trials in airway diseases were abandoned due to its short duration of action [91].

Benafentrine is a benzonaphthyridine derivative that inhibited PDE3 from guinea-pig platelets (IC_50_ 1.74 mM) and PDE4 from guinea-pig neutrophils (IC_50_ 1.76 mM) [92]. Although this compound is a relatively weak inhibitor of PDE3 and PDE4 enzymes, it was still tested in humans. Normal human subjects challenged with methacholine did not show bronchodilator activity after treatment with benafentrine at oral doses of up to 90 mg. However, benafentrine exhibited dose-dependent bronchodilation when administered by inhalation. Benafentrine has been discontinued as a drug, probably because of its short duration of action and modest efficacy [82]. Pumafentrine is a compound that has entered clinical trials and is reported to have IC_50_ values of 28 nM and 7 nM for PDE3 and PDE4, respectively [93]. This compound was expected to be in Phase-II clinical trials for the treatment of asthma but was discontinued due to failure to meet the expected duration of action. Tolafentrine is reported as a dual PDE3/4 inhibitor, but little preclinical data has been published. In a phase-II asthma trial, inhaled tolafentrine did not affect airway responses to histamine challenge, and in four patients, it caused a decrease in FEV1 greater than 10% [94], and trials for asthma were subsequently terminated [91]. ORG-30029 and ORG-20241, two compounds, were published as dual PDE3/4 inhibitors [95, 96]. Among these two, ORG-20241 inhibited pulmonary leukocyte recruitment in allergen-challenged rats [94] and was found bronchoprotective in guinea pigs when delivered by inhalation [97]. ORG-20241 was reported to have entered Phase-I trials for asthma [98], although the outcome of these trials has not been made public. Kyorin Pharmaceutical and Scottish Biomedical collaborated to develop dual PDE3/4 inhibitors, and a lead compound, KCA-965, was synthesized by coupling the pyrazo- lopyridine core of ibudilast to a PDE3-inhibitor pyrida- zinone [99]. KCA-965 was abandoned due to cardio- vascular side effects noted in dogs [100], but structure-activity relationship studies refined this molecule to design novel compounds with a range of IC_50_ values for PDE3 and PDE4. Several of these lead compounds inhibited leukocyte recruitment to the lungs after methacholine-induced airway obstruction in guinea pigs [101].

**Fig. (4):**
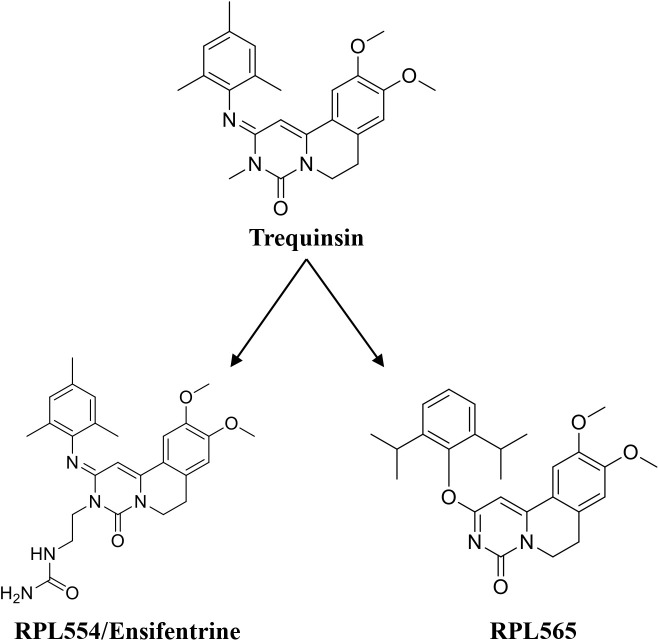
Trequinsin and its analogues (RPL554/Ensifentrine and RPL565).

Lately, two preclinical PDE3/4 dual inhibitors (RPL554 and RPL565) have been reported, which are trequinsin analogues [102] (Fig. **4**). Both compounds inhibited eosinophil recruitment in ovalbumin-sensitized guinea pigs at an oral dose of 10 mg/kg [102]. RPL554/Ensifentrine is a moderately potent PDE3 inhibitor (IC_50_ = 0.4 nM) and a weak PDE4 inhibitor (IC_50_ = 1479 nM) [102]. In a preclinical guinea pig model, inhaled RPL554 has demonstrated bronchoprotective and anti-inflammatory activities [102]. RPL554 was evaluated in two different clinical trials in patients with asthma [103] and COPD [104]. RPL554 demonstrated dose-dependent broncho- dilation in asthma patients and was well tolerated without any significant systemic adverse effects [103]. When evaluated in patients with COPD, RPL554 showed significant bronchodilator and anti-inflammatory effects [104]. Verona Pharma evaluated nebulized ensifentrine in a Phase 3 (NCT04542057) clinical study ENHANCE (Ensifentrine as a Novel Inhaled Nebulized COPD Therapy) for COPD maintenance treatment. Ensifentrine demonstrated significant improvements in lung function and reduced the rate of exacerbations in the ENHANCE-2 (NCT04542057) clinical study in patients with COPD. Another study, ENHANCE-1 (NCT04535986), successfully achieved primary and secondary endpoints, demonstrating significant improvements in lung function and reducing the rate and risk of COPD exacerbations.

RPL554 (Ohtuvayre or ensifentrine) was approved by the US Food and Drug Administration (FDA) on June 26, 2024, for the maintenance treatment of COPD in adult patients. Ohtuvayre approval was based on Phase 3 ENHANCE trials data (Verona Pharma website, June 2024).

### 2.5 PDE4 Inhibitors: Combination Therapy

In a meta-analysis, the efficacy/safety impact of triple combination ICS/LABA/LAMA with dual LABA/LAMA and ICS/LBA combinations was assessed in COPD patients after administration *via* the same inhaler device [16]. It has been well demonstrated that the addition of ICS to LABA/LAMA causes a higher incidence of pneumonia than LAMA/LABA in patients with COPD and a history of exacerbations, but it is still the preferred treatment due to the lower incidence of exacerbations and better QOL (Quality of Life) score. It has also been reported that triple therapy is superior to the LAMA/LABA combination due to the lower mortality and better dyspnea score in patients. To date, triple therapy with LAMA/LABA in patients with COPD has been reviewed in five systematic reviews [105-109]. All these reviews showed that triple therapy has a risk of pneumonia (because of ICS), but it is superior to LAMA/LABA therapy. From the safety point of view, these reviews also showed that serious adverse events were similar between triple and dual LAMA/LABA therapy. These clinical data open opportunities for combining PDE4 inhibitors or PDE3/PDE4 dual inhibitors with LAMA/LABA therapy or ICS/LAMA/LABA triple therapy since these therapies are associated with serious adverse effects, including pneumonia.

Two different clinical trials have been reported where the effect of roflumilast in addition to LABA (salmetrol) or LAMA (tiotropium) was evaluated on patients with COPD (Table **5**) [110]. The patient population included in these trials consisted of either chronic smokers or ex-smokers not using ICS at the time of randomization. In both studies, roflumilast demonstrated improvements in pre- and post-bronchodilation. The roflumilast combination with salmeterol or tiotropium exhibited an increase in pre-bronchodilator FEV1 in comparison to placebo; however, the increase was higher when combined with tiotropium (a LAMA). In both studies, a high number of withdrawals were reported in the roflumilast arm due to adverse effects.

Two more clinical studies (M2-124 and M2-125) have been reported for roflumilast. These studies aimed to investigate the effect of roflumilast on exacerbation rate and pulmonary function in patients with COPD, along with safety and tolerability evaluation [44]. In both studies, oral administration of roflumilast (500 μg, daily) reduced exacerbation frequency and improved lung function. However, 14% of patients in the roflumilast group discontinued because of class-related adverse effects.

As mentioned earlier, RPL554/Ensifentrine is a novel inhaled dual PDE3/4 inhibitor that has bronchodilator and anti-inflammatory properties. Singh *et al*. studied the short-term bronchodilator effects of RPL554 in combi- nation with other bronchodilators in COPD patients in two different clinical studies [111].

The purpose of both studies was to find out the additional bronchodilation effects of RPL554 over and above a β2-agonist or a muscarinic antagonist. In study 1, RPL554 showed clinically relevant bronchodilation, as similar effects were found after salbutamol (a SABA) and ipratropium (a SAMA) administration. The combination of RPL554 with short-acting bronchodilators (salbutamol or ipratropium) showed additional effects in terms of FEV1 when compared to monotherapies. Study 1 laid the foundation for further investigation of RPL554 in combination with long-acting bronchodilators. Therefore, tiotropium, a long-acting bronchodilator (a LAMA), was selected for study 2. Study 2 also demonstrated an additional effect of RPL554 on bronchodilation when combined with a LAMA. Overall, both studies demons- trated additional bronchodilation, reduced gas trapping, and improved airway conductance when RPL554 was administered alongside either a β2-agonist or muscarinic antagonists [111].

### 2.6 Future Prospective

Although there are hopes regarding the potential of PDE4 inhibitors for the treatment of asthma and COPD, further improvements are needed. Since PDE4 inhibitors are associated with class-related adverse effects, careful designing of compounds is required to improve the risk-to-benefit ratio. The dose-limiting toxicity of oral PDE4 inhibitors cilomilast or roflumilast has limited their use in COPD. Class-related adverse events led the researchers to develop inhaled PDE4 inhibitors to apply them directly to the site of action (lungs) to minimize systemic exposure and maximize the therapeutic effects [18]. Therefore, several PDE4 inhibitors have been developed for inhaled administration and tested in various respiratory diseases [17]. Among all tested inhaled PDE4 inhibitors, tanimilast exhibited more promising preclinical and clinical results so far. Tanimilast has shown ∼2000-fold higher drug levels in the lungs compared to the levels found in the systemic circulation. Moreover, tanimilast did not show PDE4 inhibitor class-related adverse effects and was tolerated well in a Single Ascending Dose (SAD) study. Inhaled tanimilast administration, along with ICS, LABA, and LAMA, steadily decreased inflammation in the lungs of patients with chronic bronchitis [74, 112]. These findings proved that patients with chronic bronchitis, after treatment with tanimilast, could have additional beneficial effects if they are still symptomatic even after the regular use of ICS, LABA, and LAMA [57]. Two large randomized, phase III trials (PILASTER and PILLAR) are currently going on to assess the effects of tanimilast in COPD patients [57].

Dual inhibitors of both PDE3 and PDE4 showed promise as a novel class of drugs with both anti-inflammatory and bronchodilator effects exerted by a single molecule [113]. FDA approval of RPL554 has proved that dual PDE3/4 inhibitors have additive or synergistic effects on cells involved in inflammation and broncho- dilation beyond those that an inhibitor selective for each PDE isozyme alone can achieve. Furthermore, it is now evident that inhaled RPL-554 displays synergism with muscarinic receptor antagonists in the relaxation of human airways [114-116], suggesting that the combination of a PDE3/4 inhibitor with an existing class of broncho- dilator may provide further clinical benefits than using any of these drug classes alone. Suppose synergistic effects can be optimized to improve efficacy and minimize side effect profiles without compromising duration of action. In that case, these observations are cause for optimism that dual PDE3/4 inhibitors could still emerge as a novel class of drugs for the treatment of airway diseases.

## CONCLUSION

Additional efforts are still necessary to develop more effective inhaled PDE4 inhibitors and dual PDE3/4 inhibitors. Also, more clinical data are needed to evaluate their therapeutic efficacy and safety in COPD patients in combination approaches. The data reported so far has come from shorter studies; however, longer-duration studies with a larger sample size are needed to evaluate the effect of PDE4 inhibitors (alone or in combination) on key clinical endpoints and safety parameters. The recent approval of the dual PDE3/4 inhibitor RPL554/ensifentrine sparked new hope for developing more such inhibitors. We anticipate that effective inhaled PDE4 inhibitors and dual PDE3/4 inhibitors have the potential to replace or reduce ICS and its side effects.

## Figures and Tables

**Table 1:** Approved fixed-dose combinations of LABAs and LAMAs for COPD treatment [116].

LABA/LAMA	Device	Approved Dose	Frequency of Administration
**Indacetrol/glycopyrronium**	Breezhaler	110/50 µg^a^	Once daily
	Neohaler	27.5/15.6 µg^b^	Twice daily
**Vilanterol/umeclidinum**	Ellipta	22/55 µg^a^	Once daily
		25/62.5 µg^c^	Once daily
**Formoterol/aclidinum**	Genuair	12/340 µg	Twice daily
**Olodaterol/tiotropium**	Respimat	2.5/2.5 µg^d^	Once daily

**Note:** ^a^Approved dose in Europe and Asia.

^b^Approved dose in the USA.

^c^Approved dose in USA and Japan.

^d^Two puffs once daily.

**Table 2:** Current triple fixed-dose combinations of LABAs, LAMAs and ICS for COPD treatment.

**Study Name**	**Triple Combination Used** **(ICS+LABA+LAMA) **	**No. of Subjects**	**Outcome**
**KRONOS** **NCT02497001** **(2018)**	Budesonide 640 µg Glycopyrronium 36 µg Formoterol 19.2 µg (Fixed inhaler)	639	The Phase III KRONOS trial proved the efficacy and tolerability of triple therapy relative to dual combination in patients with moderate to severe COPD irrespective of exacerbation history.
**IMPACT** **NCT02164513** **(2018)**	Fluticasone furoate 100 µg Umeclidinium 62.5 µg Vilanterol 25 µg (Fixed inhaler)	4151	The Phase III IMPACT trial showed that treatment with triple therapy significantly reduced the rate of moderate or severe COPD exacerbations and hospitalizations compared to LABA/LAMA
**TRIBUTE** **NCT02579850** **(2018)**	Beclometasone 174 µg Glycopyrronium 18 µg Formoterol 10 µg (Fixed inhaler)	764	Phase III TRIBUTE trial showed that BDP/FF/G significantly reduced the rate of moderate-to-severe exacerbations compared to LABA/LAMA, without increasing the risk of pneumonia.
**ETHOS** **NCT02465567** **(2020)**	Budesonide 640 µg Glycopyrronium 36 µg Formoterol 19.2 µg (Fixed inhaler)	2144	Phase III ETHOS trial showed that triple therapy resulted in a lower rate of moderate or severe exacerbations than dual therapy in COPD patients.

**Table 3:** Ongoing Phase III studies of tanimilast [57].

**Study ID**	**Main Objectives**	**Study Design**	**Device, Dosing Regimen, Duration**	**Treatments (Number of Subjects)**	**Population (N randomized)**	**Primary Measures**
**PILASTER** **(NCT04636801)**	Efficacy, safety, and tolerability	Multicenter, Randomised, Double-blind, Placebo-controlled, Parallel-group	NEXThaler Twice-daily (BID) 24 weeks	Tanimilast: Dose 1: 800µg BID Dose 2: 1,600 µg BID Matched placebo BID	N= 2,985 moderate to very severe COPD patients with chronic bronchitis and a history of exacerbation on maintenance with triple therapy (ICS, LABA and LAMA)	Primary efficacy variable: Rate of moderate and severe exacerbations, Main secondary variables: Time to 1st moderate or severe exacerbations, predose FEV_1 _PROs, Safety variables: AEs, AEs of special interests, vital signs, body weight, 12-lead ECGs, routine laboratory values
**PILLAR** **(NCT04636814)**	Efficacy, safety, and tolerability	Multicenter, Randomised, Double-blind, Placebo-controlled, Parallel-group	NEXThaler Twice-daily (BID) 24 weeks	Tanimilast: Dose 1: 800µg BID DOSE 2: 1,600 µg BID Roflumilast 500µg OD Matched placebo	N= 3,980 severe to very severe COPD patients with chronic bronchitis and a history of exacerbation on maintenance with triple therapy (ICS, LABA and LAMA)	Primary efficacy variable: Rate of moderate and severe exacerbations, Main secondary variables: Time to 1st moderate or severe exacerbations, predose FEV_1 _PROs, Safety variables: AEs, AEs of special interests, vital signs, body weight, 12-lead ECGs, routine laboratory values

**Table 4:** Dual PDE3/4 inhibitors entered into clinical trials for airway diseases.

**Compound**	**Chemical Structures**	**Clinical Trial Updates for the Treatment of Airway Diseases**	**Current Status**
**Zardaverine**	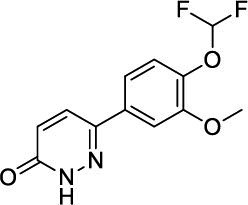	Inhaled zardaverine did not cause lung function improvements in COPD patients	Clinical trials discontinued
**Benafentrine**	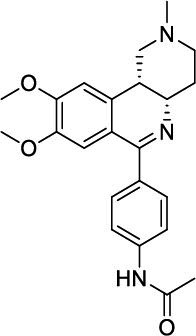	Benafentrine showed dose-dependent bronchodilation after methacholine challenge in healthy volunteers	Clinical trials discontinued
**ORG-20241**	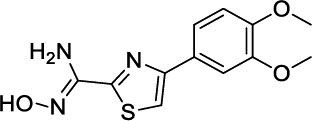	Tested in Phase I trials for Asthma but data not published	Clinical trials discontinued
**Tolafentrine**	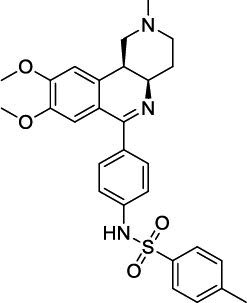	Caused decrease in FEV1 >10% in phase II asthma trials	Clinical trials discontinued
**Pumafentrine**	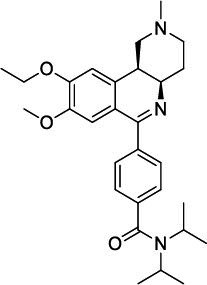	Pumafentrine entered in Phase II asthma trials but lacked efficacy	Clinical trials discontinued
**RPL-554**	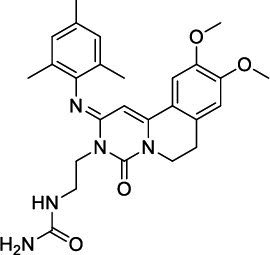	Inhibition of pulmonary leukocyte recruitment in healthy volunteers treated with aerolized LPS. Acute increase in FEV_1 _in patients with asthma and patients with COPD.	Completed Phase II trials and currently in Phase III trials (clinicaltrials.gov; NCT04542057)

**Table 5:** A summary of the most important clinical trials with PDE4 inhibitors and long-acting bronchodilators in COPD.

**Duration of Treatment**	**Treatment Groups**	**Severity**	**Endpoints**	**Results**
**24 Weeks (M2-127)**	Placebo (467/385) Roflumilast 0.5mg OD (466/359) Salmeterol both groups	50%	Exacerbations: (GOLD IV) Prebroncodilator FEV_1 _Postbroncodialtors FEV_1 _ Mean rate of exacerbations (mild, moderate, severe) Dyspnea index	36% Lower 49ml increase over placebo/salmeterol 60ml increase over placebo/salmeterol NS *vs* placebo/salmeterol NS *vs* placebo/salmeterol
**24 Weeks (M2-128)**	Placebo (372/333) Roflumilast 0.5mg OD (371/309) Tiotropium both groups	50%	Prebroncodilator FEV_1 _Postbroncodialtors FEV_1 _ Mean rate of exacerbations (mild, moderate, severe) Dyspnea index (TDI focal score/change in SOBQ)	80ml increase over placebo/tiotropium 81ml increase over placebo/tiotropium NS *vs* placebo/tiotropium 0.4units/2.6 units significantly better with roflumilast

## LIST OF ABBREVIATIONS

COPD: = Chronic Obstructive Pulmonary Disease
ICS: = Inhaled Corticosteroids
FR: = Fixed Ratio
